# Periconception Maternal Vitamin D Status on Nausea and Vomiting Symptoms in Early Pregnancy Among Women with a History of Pregnancy Loss

**DOI:** 10.3390/nu18040692

**Published:** 2026-02-21

**Authors:** Zeina M. Alkhalaf, Sunni L. Mumford, Enrique F. Schisterman, Robert M. Silver, Marie E. Thoma

**Affiliations:** 1 Department of Family Science, School of Public Health, University of Maryland, College Park, MD 20742, USA; 2 Epidemiology Branch, Division of Intramural Population Health Research, Eunice Kennedy Shriver National Institute of Child Health and Human Development, National Institutes of Health, Bethesda, MD 20892, USA; 3 Department of Biostatistics, Epidemiology and Informatics and Department of Obstetrics and Gynecology, Perelman School of Medicine, University of Pennsylvania, Philadelphia, PA 19104, USA; 4 Department of Obstetrics and Gynecology, University of Utah, Salt Lake City, UT 84132, USA

**Keywords:** pregnancy, vitamin D, preconception, nutrition, pregnancy outcomes

## Abstract

Background/Objectives: Sufficient preconception vitamin D may promote robust implantation and higher human chorionic gonadotropin (hCG) levels, potentially increasing nausea and vomiting in pregnancy. We assessed associations between maternal serum 25-hydroxyvitamin D (25(OH)D) at both preconception and 8 weeks’ gestation with nausea and vomiting during early pregnancy. We hypothesized that women with sufficient vitamin D status or those who improved their levels in early gestation, would have higher odds of nausea and vomiting compared to women who were deficient or insufficient. Methods: This secondary analysis of the randomized EAGeR Trial included women with 1–2 prior pregnancy losses and 25(OH)D measured at preconception (*n* = 774) and 8 weeks’ gestation (N = 641). Nausea and vomiting were captured via medical records and daily symptom diaries. 25(OH)D was categorized as deficient (≤20 ng/mL), insufficient (21–29 ng/mL), or sufficient (≥30 ng/mL). Logistic regression and generalized estimating equations (GEE) estimated associations. Results: Women who improved from deficient/insufficient preconception to sufficient by 8 weeks had higher odds of nausea and vomiting in early pregnancy compared to those remaining sufficient (aOR: 1.71; 95% CI: 1.12, 2.61). Conversely, those remaining deficient/insufficient (aOR: 0.34; 95% CI: 0.20, 0.60) or declining to deficiency (aOR: 0.44; 95% CI: 0.22, 0.87) had lower odds. In longitudinal models, deficiency was associated with lower odds of daily vomiting (aOR: 0.54; 95% CI: 0.28, 1.04), though estimates were imprecise. Conclusion: Dynamic changes in vitamin D status from preconception to early pregnancy appear to be associated with nausea and vomiting in early pregnancy. Improvement of sufficiency increased emesis odds, while persistent deficiency correlated with fewer symptoms. These findings suggest vitamin D may be associated with nausea and vomiting through hormonal or placental signaling mechanisms in early gestation.

## 1. Introduction

Nausea is a common early pregnancy symptom, affecting approximately 50–70% of pregnant women, with the onset of symptoms occurring between 2 and 4 weeks of gestation [1]. It has been suggested that successful implantation and healthy placental function result in higher secretion of human chorionic gonadotropin (hCG), which may manifest clinically as a more heightened nausea response [1]. Additionally, prior research indicates that nausea during early pregnancy may be associated with a lower risk of miscarriage, preterm birth, low birth weight (LBW), and perinatal death [2,3,4].

Although nausea is thought to be indicative of a more robust implantation response, underlying mechanisms for the development of nausea or emesis (vomiting) in pregnancy remain unclear. Greater nutrient stores around the time of implantation have been linked to increased pregnancy and live birth rates and, accordingly, may present clinically as a heightened nausea response [1,4,5,6]. Accordingly, the directionality of these associations are difficult to discern in the absence of measures in early pregnancy, as nutrient depletion may cause or be caused by emesis or the inability to take in nutrients from food sources [7,8]. However, because vitamin D is primarily obtained through UV exposure and supplementation rather than food intake, serum concentrations may be less influenced by short-term reductions in oral intake due to nausea or vomiting [9,10].

Vitamin D, which is a secosteroid hormone, has been postulated to have an impact on early critical periods of implantation and may lead to adverse pregnancy outcomes if not above recommended sufficiency levels (>30 ng/mL) by the Endocrine Society [11,12,13,14,15,16]. If sufficient levels of vitamin D are present in trophoblast cells and help form the placenta successfully, this may lead to a robust implantation process, increasing levels of hCG, which is associated with a higher likelihood of nausea in pregnancy [1,17,18]. Additional to sufficient vitamin D levels leading to healthy placentation processes, sufficient vitamin D in early pregnancy modulates inflammation, regulates hormone production, and supports nutrient transfer to the placenta [19,20,21]. Thus, continuous sufficient vitamin D throughout the pregnancy could support placental health and sufficient hormone regulation [20]. If any disruptions occur during the preconception and early pregnancy period, placental dysfunction may occur, and a robust nausea response may be diminished and/or result in adverse pregnancy outcomes [22,23,24,25,26,27]. Therefore, like folic acid, achieving sufficient vitamin D levels prior to or during the periconceptual period may be essential for optimal placental development [28]. Importantly, largely because vitamin D status is derived from UV exposure and supplementation rather than diet alone, serum concentrations are less likely to be influenced by short-term reductions in nutrient intake due to symptoms such as nausea and vomiting, unless it becomes persistent throughout the pregnancy period. In recognition of its importance in the early pregnancy period, the Endocrine Society has updated their vitamin D and pregnancy guidelines by recommending universal vitamin D supplementation to pregnant women of 2500 IU per day to ensure sufficient levels are achieved and maintained during pregnancy [29].

Therefore, the objective of this study was to assess the association between maternal serum 25-hydroxyvitamin D (25(OH)D) concentrations at preconception and 8 weeks’ gestation and nausea or vomiting during placentation and early pregnancy.

## 2. Materials and Methods

The proposed research utilizes data from the Effects of Aspirin in Gestation and Reproduction (EAGeR) trial, a multisite, prospective, double-blind, block-randomized, placebo-controlled clinical trial designed to evaluate the effect of low-dose aspirin (LDA) on live birth in healthy women with regular menstrual cycles and 1–2 prior pregnancy losses [30]. A total of 1228 healthy women between 18 and 40 years of age were enrolled in the trial and could not have received fertility treatments prior to or during their enrollment or have a prior diagnosis of infertility. The institutional review boards at each study site (Salt Lake City, Utah; Denver, Colorado; Buffalo, New York; Scranton, Pennsylvania) and the data coordinating center approved the protocol for the trial. All participants provided their written consent prior to enrolling in the study. The trial was registered with ClinicalTrials.gov (#NCT00467363).

### 2.1. Study Design and Population

**Figure 1:**
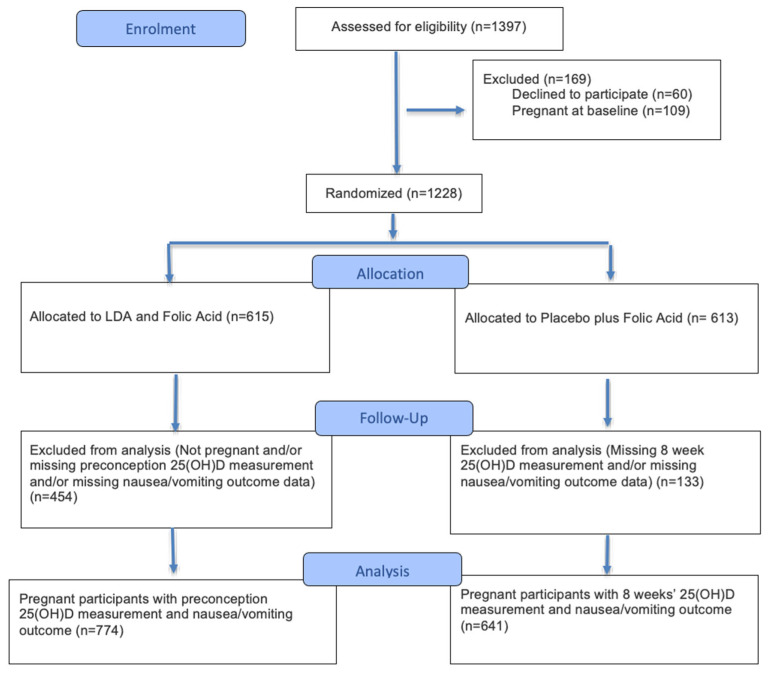
CONSORT flow diagram of participant enrollment, allocation, follow-up, and secondary analysis of the EAGeR Trial.

This is a secondary analysis of the EAGeR Trial that included individuals who became pregnant and had measured serum 25(OH)D levels at preconception (n = 774) and 8 weeks’ gestation (n = 641) and no missing data on nausea or vomiting measures (Figure 1). Differences in analytic sample size across time points reflect the longitudinal design and timing of pregnancy progression rather than the predefined exposure subgroup allocation. Analytic samples were restricted to participants who had measured vitamin D and nausea/vomiting symptoms data at both preconception and 8 weeks’ gestation with inverse probability weighting to address potential selection bias related to becoming pregnant and follow-up. Women were followed for up to 6 cycles while attempting pregnancy, and throughout pregnancy if they conceived. The median time to conception was approximately 3 months. Pregnancy status was determined via positive urine hCG pregnancy tests (Quidel Quickvue, Quidel Corporation, San Diego, CA, USA), conducted at home or in the clinic at the time of expected menses.

### 2.2. Vitamin D Assessment

Serum samples were collected at baseline prior to randomization to low-dose aspirin (LDA) or a placebo and at 8 weeks’ gestation among women who conceived. The serum samples were collected during the study from 2007 to 2012 and stored at −80 °C until used for analysis, which was conducted from 2014 to 2015 [30]. Thus, storage duration ranged from approximately 2 to 8 years, depending on the collection date. Combined concentrations of 25-hydroxyvitamins D2 and D3 (25(OH)D) were measured using the 25(OH)D ELISA solid phase sandwich enzyme immunoassay (BioVendor R&D, Ashville, NC, USA), which has been validated previously [31].

The Endocrine Society has established vitamin D cutoffs that have been used in this analysis to inform clinical interpretation [32]. Locally weighted scatterplot smoothing (LOWESS) was used to evaluate the functional form of the association between continuous 25(OH)D status and nausea and vomiting outcomes, which suggested a potential non-linear relationship and supported categorical exposure modeling. Vitamin D categories were classified as 25(OH)D deficient (≤20 ng/mL), insufficient (21–<30 ng/mL), or sufficient (≥30 ng/mL) at preconception and 8 weeks’ gestation [32]. Change in vitamin D status between preconception and 8 weeks’ gestation was assessed by combining deficient and insufficient vitamin D together and categorizing the change as improved (deficient/insufficient to sufficient), declined (sufficient to deficient/insufficient), no change (remained deficient/insufficient), and no change (remained sufficient).

### 2.3. Outcome Measures

Although participants reported a range of early pregnancy symptoms, the present analysis focused specifically on nausea and vomiting, which were predefined outcomes based on biologic hypotheses related to vitamin D and early placental signaling. Additionally, outcomes of nausea and vomiting reflect symptom-based measures of nausea and vomiting and do not represent clinical diagnosis of hyperemesis gravidarum.

#### 2.3.1. Nausea/Emesis Chart Abstractions

Nausea/emesis (vomiting) was assessed using check-box questions via medical chart abstractions completed by study staff. Following study completion, case report forms and open-ended questions completed through questionnaires and medical records were independently reviewed by two board-certified reproductive endocrinologists as well as a perinatal epidemiologist [33]. Medical record abstraction was used to capture clinical documentation of nausea or emesis (vomiting) present during the pregnancy (i.e., ever medically documented during the pregnancy).

#### 2.3.2. Nausea/Vomiting Daily Diaries

Time-varying outcomes of nausea and vomiting symptoms were also collected by self-reporting through daily diaries during the first two preconception cycles, and if they conceived, through the first 4 weeks of clinically confirmed pregnancy (up to 8 weeks’ gestational age). Daily diary questions assessing symptom severity of nausea and vomiting were “Please report any nausea or vomiting that you have experienced today. Record these symptoms regardless of the reason, if none, please enter 0.” Responses were categorized as follows: 1 = none, 2 = nausea, 3 = vomiting once per day or more. Previous studies have validated self-reported nausea and have found reasonable accuracy between the self-reported data and clinical presentations [34]. Nausea and vomiting categories were defined at biweekly intervals between 3 and 4 weeks, 5 and 6 weeks, and 7 and 8 weeks of gestation and were further divided into binary groups at each time point and classified for analysis as reporting any nausea or vomiting (vs. none), and by symptom type as nausea only (vs. none), or vomiting once per day or more (vs. none). Analyses of daily diary data were conducted separately from the medical chart abstraction data analyses.

### 2.4. Covariates

Participants were enrolled in the EAGeR Trial, which excluded women with any major medical conditions or reproductive disorders that may influence pregnancy outcomes or vitamin D metabolism. Full clinical eligibility details are described elsewhere [30]. At baseline, participants completed standardized questionnaires assessing demographic characteristics (e.g., age, self-identified race/ethnicity, education, employment, income), lifestyle factors (e.g., physical activity, alcohol consumption, multivitamin use), and reproductive history (e.g., parity). Anthropometric measurements (e.g., height, weight) were measured by trained study staff using standardized protocols to calculate body mass index (BMI kg/m^2^). The selection of covariates for adjustment was informed by prior literature and guided by directed acyclic graphs (DAGs) [35,36]. Additionally, aspirin/placebo, the assigned treatment in this trial, was considered as a confounder in this analysis, as previously applied in a study of vitamin D and pregnancy loss using these data [37].

### 2.5. Statistical Analysis

#### 2.5.1. Descriptive Analyses

Descriptive baseline characteristics for women in the EAGeR Trial in this analysis by preconception 25(OH)D status were examined using chi-square tests or ANOVA for categorical or continuous variables, respectively.

#### 2.5.2. Logistic Regression Models

Unadjusted and adjusted odds ratios for the association between the change in preconception and 8 weeks’ gestation serum 25(OH)D levels and nausea/vomiting as reported on medical records were estimated using logistic regression models with robust standard errors. Models were adjusted for relevant confounders, as determined by Directed Acyclic Graphs (DAGs), which included all sociodemographic factors: age, season, income, race, education, parity, employment; and lifestyle factors: smoking, exercise, alcohol, treatment assignment, vitamin D, use of multivitamins, including BMI.

We applied inverse probability weights to account for selection that may occur by only including women who had a pregnancy (i.e., excludes women who did not become pregnant during the study period, which may also be related to the exposure under study). The inverse probability weights were derived from models that included covariates associated with the probability of being pregnant, including age, smoking, season, exercise, income, race, education, alcohol, parity, treatment assignment, employment, vitamin D, vitamin use, and BMI [35,36].

Distributional characteristics of serum 25(OH)D were examined visually and LOWESSs plots were used to assess functional form; because logistic and GEE models do not assume normally distributed predictors, formal normality testing was not required.

#### 2.5.3. Generalized Estimating Equations (GEE) Regression Models

To account for repeated measures of symptom severity of nausea and vomiting across biweekly intervals in the first 8 weeks of pregnancy at 3 time points (3–4, 5–6, and 7–8 weeks), we used generalized estimating equations (GEE) with a binomial family and logit link to estimate odds ratios (ORs) and 95% confidence intervals with an unstructured correlation matrix. 25(OH)D levels at preconception were applied to the first interval (3–4 weeks) and 25(OH)D levels measured at 8 weeks were applied to the last time interval (7–8 weeks gestation). For weeks 5–6, an average of the preconception and 8 weeks’ gestation 25(OH)D level was imputed. Models were adjusted for the same set of covariates applied above. Separate GEE logistic regression models were estimated for each outcome, each operationalized as a dichotomous variable comparing symptomatic women to those who reported no symptoms: (1) any nausea or vomiting (vs. none), (2) nausea only (vs. none), and (3) vomiting once per day or more (vs. none). Inverse probability weights to account for potential selection bias of becoming pregnant were included as described above.

### 2.6. Sensitivity Analyses

To account for nausea/vomiting tending to be associated with a reduced risk of pregnancy loss and assess the robustness of our findings, we compared results from our main analyses to analyses restricted to women who achieved a live birth (Appendix A Table A1) [2]. Given selection bias may result from also restricting to pregnancies that survive to a live birth, additional inverse probability weights were applied to account for this potential bias [35,36]. All inverse probability weight models accounted for age, smoking, season, exercise, income, race, education, alcohol, parity, treatment assignment, employment, vitamin D, vitamin use, and BMI [35,36]. All analyses were performed using STATA version 17.0.

## 3. Results

### 3.1. Characteristics of Participants

Participant characteristics by preconception 25-hydroxyvitamin D (25(OH)D) status are presented in Table 1. Mean maternal age was similar across categories (sufficient: 28.7 ± 4.4 years; insufficient: 28.7 ± 4.6 years; deficient: 28.5 ± 5.3 years). However, several factors varied by vitamin D status:

- BMI: Women with 25(OH)D deficiency had a significantly higher mean BMI (30.50 ± 8.63 kg/m^2^) compared to those with sufficient (24.47 ± 5.1 kg/m^2^) or insufficient (26.9 ± 6.3 kg/m^2^) levels.
- Physical Activity: 40% of the deficient group reported low physical activity, compared to 23% of the sufficient group.
- Education: 81% of women with deficiency had attained education beyond high school, versus 90% in the sufficient group.
- Seasonality: Season of measurement differed significantly; in the deficient group, 38% were measured in the fall and 9% in the summer. In contrast, the sufficient group was more evenly distributed between fall (28%) and summer (26%).
- Other sociodemographic characteristics were broadly similar across 25(OH)D categories.

### 3.2. Vitamin D Dynamics and Risk of Nausea and Emesis

In logistic regression models evaluating nausea/emesis documented in medical records (Table 2), the distribution of 25(OH)D status change from preconception to 8 weeks’ gestation was categorized as

1. Remained sufficient: 207 (38.6%)
2. Declined to deficient/insufficient: 160 (29.8%)
3. Improved to sufficient: 105 (19.6%)
4. Remained deficient/insufficient: 65 (12.1%)

Women who improved their status from deficient/insufficient at preconception to sufficient at 8 weeks had significantly higher odds of nausea/emesis (aOR 1.71; 95% CI: 1.12, 2.61) compared to those who remained sufficient. Conversely, lower odds of symptoms were observed in women whose levels declined (aOR 0.44; 95% CI: 0.22, 0.87) or remained persistently deficient/insufficient (aOR 0.34; 95% CI: 0.20, 0.60).

In a sensitivity analysis restricted to live births (n = 557), the distribution of 25(OH)D changes remained similar. However, the association for the “improved” group was attenuated (aOR 0.82; 95% CI: 0.32, 2.07). The persistently deficient group maintained 40% lower odds of symptoms, though results were less precise (aOR 0.64; 95% CI: 0.25, 1.63) (Appendix A Table A1).

### 3.3. Longitudinal Analysis of Daily Symptom Severity

Longitudinal GEE analyses of daily symptom diaries (weeks 3–8) are summarized in Table 3. Compared to women with sufficient status, deficient 25(OH)D was associated with lower odds of any nausea or vomiting (aOR 0.65; 95% CI: 0.40, 1.06). When evaluating specific symptoms:

- Deficient 25(OH)D was associated with decreased odds of vomiting one time per day (aOR 0.54; 95% CI: 0.28, 1.04).
- Deficient 25(OH)D was associated with lower odds of nausea only versus no symptoms (aOR 0.84; 95% CI: 0.63, 1.12).

Results for women with insufficient 25(OH)D levels were similar to those with sufficient status for all outcomes, including any nausea/vomiting (aOR 0.86; 95% CI: 0.65, 1.15) and vomiting one time per day (aOR 1.12; 95% CI: 0.80, 1.56). Trends remained consistent in the live birth restricted sample, though precision decreased due to reduced sample size (Appendix A Table A2). Distribution of vitamin D and nausea and vomiting symptoms for those with a pregnancy loss versus a live birth can be found in Appendix A Table A3.

## 4. Discussion

In our study among healthy women with prior history of one to two pregnancy losses, our findings suggest that higher levels of 25(OH)D may be associated with symptoms of nausea and vomiting, represented by both clinically documented and self-reported symptoms during pregnancy [1,2]. Because fewer participants were classified as 25(OH)D deficient (<20 ng/mL), estimates for this subgroup may be less precise and should be interpreted cautiously. However, several sociodemographic and behavioral characteristics differed across vitamin D categories, suggesting that 25(OH)D status may reflect underlying lifestyle and sociodemographic patterns within the cohort. Similar directional patterns were observed using both nausea and vomiting as documented in the medical chart abstraction and by symptom severity reported in the daily diaries, indicating that findings were consistent across assessment methods. In the full sample that included pregnancy losses, we found that women who improved their vitamin D status from deficient or insufficient at preconception to sufficient at 8 weeks’ gestation had higher odds of experiencing medically documented nausea or emesis symptoms compared to those who remained sufficient at both time points. Women who remained deficient/insufficient or declined in their 25(OH)D status to deficiency had lowers odds of experiencing these symptoms.

In the longitudinal GEE models of symptom severity in the daily diaries across 3–8 weeks’ gestation, deficient 25(OH)D status was associated with a reduced odds of time-varying nausea and vomiting, in particular among those experiencing vomiting once or more per day, although the results were imprecise. After restriction to live births, these results were attenuated but still showed a reduction in odds of clinical symptoms, suggesting that the association between deficient vitamin D and pregnancy symptoms of nausea/emesis may be related to both early pregnancy losses and other mechanisms that may be associated with a more robust implantation response. This pattern corroborated previous studies that have indicated the presence of nausea and vomiting during pregnancy may suggest healthy placental signaling and hormonal receptivity during early pregnancy, and that vitamin D deficiency during this period may impair these biological processes [7,17,38,39].

Vitamin D deficiency may lead to placental dysfunction due to the disruption of hormones that help maintain a pregnancy and support endometrial receptivity and implantation of the uterus, such as that of estrogen and progesterone [39,40,41,42,43,44]. Specifically, trophoblasts are located in the outer layers of endometrial cells and help the embryo implant successfully and then form the placenta [45]. These trophoblasts then support endometrial receptivity via vitamin D receptors in the uterus by providing an anti-inflammatory environment for successful implantation and placentation [39,40,41,42].

There is limited information on the relationship between preconception and early gestation maternal vitamin D and clinical experiences of nausea and vomiting in early pregnancy. Our study, however, is consistent with prior work from this cohort which found that sufficient preconception 25(OH)D may increase the odds of live birth, which may correlate with robust implantation [37]. In addition, a previous IVF study measured preconception vitamin D status and found sufficient preconception vitamin D increased the incidence of pregnancy, also suggesting a robustness in implantation and placentation [46].

The biological pathways for the role of preconception, early pregnancy period and vitamin D in increasing the risk of nausea and vomiting are likely multifactorial. This study is suggestive of the importance of sufficient maternal vitamin D stores prior to conception and the incidence of nausea and vomiting, which has been associated with an increase in the production of hCG and aids in maintenance of the pregnancy [2,7,47]. Future studies with larger sample sizes are needed to assess the association between preconception vitamin D on severe nausea and vomiting during pregnancy.

A major strength of this study is the availability of vitamin D measurements at both preconception and 8 weeks’ gestation, which are critical time periods for implantation and placental development. The daily diaries captured nausea and vomiting symptoms from as early as 3–4 weeks gestation, often before women find out they are pregnant, which allows for precise and prospective symptom tracking over time.

This study has several limitations, with one being the sample size was limited for examining more detailed groupings or interactions. Nausea and vomiting were assessed through both medical records and self-reported daily diaries, enabling time-varying longitudinal analyses. The consistency of findings across different types of data collection sources, each with different potential biases, strengthens our findings. Our cohort included women with one to two prior pregnancy losses and that may restrict generalizability to the general obstetric population. However, the prevalence of nausea symptoms observed in this cohort was similar to that reported in general obstetric populations.

For the GEE analysis, we inferred vitamin D levels at 5–6 weeks’ gestation as the average between preconception and 8 weeks’ gestation, due to a lack of direct measurement, and variation in timing may affect accuracy. While the persistently deficient group continued to demonstrate reduced odds of nausea and vomiting, precision was limited due to a small sample size. Analyses that were restricted to live births showed similar, but attenuated, patterns of association. Additionally, women who improved their 25(OH)D status from deficient at preconception to sufficient at 8 weeks’ gestation may be more likely to have tolerated supplements, or had more sun exposure during that time period, which may explain the higher odds of symptoms in this group, rather than a biologic effect of 25(OH)D on symptom severity. Finally, detailed dietary intake and medication use beyond the eligibility screenings were not collected in EAGeR.

## 5. Conclusions

The biologic pathways underlying the associations observed between preconception and early gestation maternal serum 25(OH)D on nausea and vomiting are most likely multifactorial, and this study is suggestive of deficient maternal 25(OH)D nutrient stores prior to conception being associated with reduced odds of experiencing nausea or vomiting. Sufficient 25(OH)D nutrient stores during the preconception and early gestation period are important and may be an indicator of a more robust implantation/placentation and therefore a healthy pregnancy. Future studies are needed with larger sample sizes to assess the association between 25(OH)D and nausea and vomiting in the preconception and early gestation period in more diverse populations.

## Figures and Tables

**Table 1:** Descriptive characteristics of women in the EAGeR Trial who became pregnant by preconception maternal serum 25(OH)D status (N = 774).

	25(OH)D Sufficient (≥30 ng/mL)	25(OH)D Insufficient (21–29 ng/mL)	25(OH)D Deficient (<20 ng/mL)
N	392 (50.6%)	285 (36.8%)	97 (12.5%)
Age, years	n (%)	n (%)	n (%)
Mean ± SD	28.7 ± 4.4	28.7 ± 4.6	28.5 ± 5.3
18–24.9	95 (24)	59 (21)	22 (23)
25–29.9	148 (38)	126 (44)	41 (42)
30–34.9	97 (25)	76 (27)	23 (24)
35–40.9	52 (13)	24 (8)	11 (11)
* BMI, kg/m^2^			
Mean ± SD	24.5 ± 5.1	26.9 ± 6.3	30.5 ± 8.6
Underweight < 18.5	15 (4)	12 (4)	3 (3)
Normal ≥ 18.5 and <25	247 (64)	132 (46)	30 (31)
Overweight ≥ 25 and <30	81 (21)	88 (31)	17 (18)
Obese ≥ 30	45 (12)	52 (18)	46 (48)
* Self-reported Race			
White	387 (99)	278 (98)	81 (84)
Non-White	5 (1)	7 (2)	16 (16)
Education			
≤High School	40 (11)	23 (8)	18 (19)
>High School	352 (90)	262 (92)	79 (81)
Income, n			
≥$100,000	151 (39)	130 (46)	33 (34)
$75,000–$99,999	64 (16)	40 (14)	8 (8)
$40,000–$74,999	65 (17)	34 (12)	13 (13)
$20,000–$39,999	86 (22)	64 (22)	32 (33)
≤$19,999	26 (7)	17 (6)	11 (11)
Employment			
Yes	92 (24)	79 (28)	28 (30)
No	295 (76)	203 (72)	65 (70)
Vitamin Use			
No Folic Acid–No Vitamins	29 (7)	12 (4)	7 (7)
No Folic Acid–Yes Vitamins	66 (17)	41 (14)	10 (10)
Yes Folic Acid–Yes Vitamins	297 (76)	232 (81)	80 (82)
Smoking			
Never	345 (89)	190 (90.5)	60 (88.2)
<6 times/week	32 (8)	12 (5.7)	6 (8.8)
Daily	12 (3)	8 (3.8)	2 (2.9)
Season			
Fall (Sep-Nov)	108 (28)	71 (25)	37 (38)
Winter (Dec-Feb)	76 (19)	65 (23)	27 (28)
Spring (Mar-May)	105 (28)	86 (30)	24 (25)
Summer (Jun-Aug)	103 (26)	63 (22)	9 (9)
* Exercise			
Low	89 (23)	74 (26)	39 (40)
Moderate	173 (44)	110 (39)	39 (40)
High	130 (33)	101 (35)	19 (20)
Number of previous pregnancy losses			
1	229 (59)	176 (62)	65 (67)
2	163 (42)	109 (38)	32 (33)
Alcohol Intensity			
Never	238 (61)	210 (75)	69 (71)
Sometimes	141 (36)	61 (22)	28 (29)
Often	9 (2)	10 (4)	0 (0)
Treatment assignment			
Placebo	179 (46)	144 (51)	48 (49)
Low-Dose Aspirin	213 (54)	141 (49)	49 (51)

* Non-white participants include American Indian/Alaska Native, Asian, Native Hawaiian or Other Pacific Islander, Black or African American, more than one Race, Unknown or Not Reported. * BMI—Body Mass Index. * Exercise level was measured through the International Physical Activity Questionnaire assessed the level of physical activity as low, moderate, and high.

**Table 2:** Association between the change in preconception and 8-week 25(OH)D status and medically documented nausea/emesis (vomiting) restricted to pregnancy: EAGeR Trial.

Restricted to Pregnancy			
Change in 25(OH)D from Preconception to 8 Weeks’ Gestation	N = 747	Unadjusted ^1^ OR (95% CI)	Adjusted ^2^ OR (95% CI)
Improved: Deficient/Insufficient to Sufficient	105 (19.6)	1.69 (1.14, 2.50)	1.71 (1.12, 2.61)
Declined: Sufficient to Deficient/Insufficient	65 (12.1)	0.44 (0.23, 0.84)	0.44 (0.22, 0.87)
No Change: Deficient/Insufficient	160 (29.8)	0.40 (0.24, 0.66)	0.34 (0.20, 0.60)
No Change: Sufficient	207 (38.6)	ref.	ref.

^1^ Unadjusted for covariates and weighted to control for potential selection bias introduced by restricting to a sample of pregnancy. ^2^ Adjusted for all sociodemographic and lifestyle covariates which included age, smoking, season, exercise, income, race, education, alcohol, parity, aspirin, employment, vitamins, and BMI and weighted to control for potential selection bias introduced by restricting to a sample of pregnant women.

**Table 3:** Odds ratio and 95% CI between time-varying maternal serum 25(OH)D level and nausea and vomiting episodes from 3 to 8 weeks gestation among women who became pregnant in the EAGeR Trial.

		Unadjusted–Model 1 ^1^ OR (95% CI)	Adjusted–Model 2 ^2^ OR (95% CI)	Adjusted–Model 3 ^3^ OR (95% CI)
Any Nausea or Vomiting (vs. None)	N = 1723			
Deficient		0.70 (0.46, 1.07)	0.77 (0.49, 1.23)	0.65 (0.40, 1.06)
Insufficient		0.92 (0.70, 1.20)	0.91 (0.69, 1.20)	0.86 (0.65, 1.15)
Sufficient		ref	ref	ref
Nausea Only (vs. None)	N = 1562			
Deficient		0.67 (0.43, 1.03)	0.76 (0.47, 1.21)	0.65 (0.40, 1.07)
Insufficient		0.88 (0.67, 1.16)	0.88 (0.66, 1.17)	0.84 (0.63, 1.12)
Sufficient		ref	ref	ref
Vomiting once per day or more (vs. None)	N = 498			
Deficient		0.87 (0.48, 1.60)	0.69 (0.35, 1.35)	0.54 (0.28, 1.04)
Insufficient		1.29 (0.92, 1.82)	1.19 (0.84, 1.69)	1.12 (0.80, 1.56)
Sufficient		ref	ref	ref

N corresponds to longitudinal observations (not women). Three time windows are assessed: 3–4 weeks’ gestation, 5–6 weeks, 7–8 weeks. Time-varying 25(OH)D: 3–4 weeks utilizes preconception 25(OH)D levels, 5–6 weeks utilizes the average between preconception and 8 weeks 25(OH)D, and 7–8 weeks utilizes the 8 weeks 25(OH)D levels. ^1^ Unadjusted model and weighted to control for potential selection bias introduced by restricting to a sample of pregnancy. ^2^ Adjusted for all sociodemographic covariates which include age, exercise, income, race, education, parity, employment, and season and weighted to control for potential selection bias introduced by restricting to a sample of pregnant women. ^3^ Adjusted for all sociodemographic and lifestyle covariates which included age, exercise, income, race, education, parity, employment, alcohol, vitamins, aspirin, and BMI and weighted to control for potential selection bias introduced by restricting to a sample of pregnant women.

## Data Availability

The data underlying this article have been provided by the *Eunice Kennedy Shriver* National Institute of Child Health and Human Development (NICHD), National Institutes of Health under a data use agreement. We do not have permission to share it with other individuals, within or outside the primary author’s institution, or with commercial enterprises. Data from the EAGeR study are available to approved researchers through the data use agreement. Information about the study and data are available here: https//www.nichd.nih.gov/about/org/dir/dph/officebranch/eb/effects-aspirin (accessed on 05/02/2026). Data used in this study were created using pre-existing questionnaires, and derived variables from this data were previously returned to NICHD contacts for this project.

## Abbreviations

The following abbreviations are used in this manuscript:

GEE	Generalized Estimating Equations
25(OH)D	25-Hydroxyvitamin D
HCG	Human Chorionic Gonadotropin
EAGeR	Effects of Aspirin in Gestation and Reproduction
LBW	Low Birth Weight
DAG	Directed Acyclic Graphs
OR	Odds Ratio
aOR	Adjusted Odds Ratio
BMI	Body Mass Index
LDA	Low-Dose Aspirin

## Appendix A

### Appendix A.1

**Table A1:** Association Between the Change in Preconception and 8-week 25(OH)D Status and Medically Documented Nausea/Emesis (Vomiting) Restricted to Live Birth: EAGeR Trial.

Restricted to Live Birth			
Change in 25(OH)D from Preconception to 8 Weeks Gestation	N = 557	Unadjusted ^1^ OR (95% CI)	Adjusted ^2^ OR (95% CI)
Improved: Deficient/Insufficient to Sufficient	99 (19.1)	0.88 (0.34, 2.24)	0.82 (0.32, 2.07)
Declined: Sufficient to Deficient/Insufficient	61 (11.8)	1.15 (0.43, 3.06)	1.01 (0.33, 3.08)
No Change: Deficient/Insufficient	156 (30.1)	0.77 (0.32, 1.82)	0.64 (0.25, 1.63)
No Change: Sufficient	202 (39.0)	ref	ref

^1^ Unadjusted for covariates and weighted to control for potential selection bias introduced by restricting to a sample of pregnancy. ^2^ Adjusted for all sociodemographic and lifestyle covariates which included age, smoking, season, exercise, income, race, education, alcohol, parity, aspirin, employment, vitamins, and BMI and weighted to control for potential selection bias introduced by restricting to a sample of pregnant women.

### Appendix A.2

**Table A2:** Odds Ratio and 95% CI between Time-Varying Maternal Serum 25(OH)D level and Nausea/Vomiting Episodes from 3–8 weeks Gestation Restricted to Live Birth: EAGeR Trial.

EAGeR Generalized Estimating Equations Regression Models for 25(OH)D and Nausea/Vomiting				
		Unadjusted- Model 1 ^1^ OR (95% CI)	Adjusted- Model 2 ^2^ OR (95% CI)	Adjusted- Model 3 ^3^ OR (95% CI)
**Any Nausea or Vomiting (vs. None)**	N = 1468			
Deficient		0.68 (0.42, 1.10)	0.73 (0.43, 1.24)	0.60 (0.35, 1.05)
Insufficient		0.94 (0.69, 1.27)	0.93 (0.68, 1.27)	0.89 (0.65, 1.23)
Sufficient		ref	ref	ref
**Nausea Only (vs. None)**	N = 1323			
Deficient		0.62 (0.38, 1.01)	0.69 (0.40, 1.18)	0.58 (0.33, 1.02)
Insufficient		0.89 (0.65, 1.21)	0.89 (0.65, 1.22)	0.86 (0.62, 1.19)
Sufficient		ref	ref	ref
**Vomiting once per day or more than once per day (vs. None)**	N = 402			
Deficient		1.08 (0.57, 2.06)	0.88 (0.44, 1.78)	0.64 (0.33, 1.26)
Insufficient		1.35 (0.95, 1.94)	1.21 (0.85, 1.71)	1.12 (0.80, 1.57)
Sufficient		ref	ref	ref

N corresponds to longitudinal observations (not women). 3 time windows of interest (3–4 weeks gestation, 5–6 weeks, 7–8 weeks). Time Varying 25(OH)D: 3–4 weeks utilizes preconception 25(OH)D levels, 5–6 weeks utilizes the average between preconception and 8 week 25(OH)D, and 7–8 weeks utilizes the 8 week 25(OH)D levels. ^1^ Unadjusted model and weighted to control for potential selection bias introduced by restricting to a sample of Live Birth. ^2^ Adjusted for all sociodemographic covariates which include age, exercise, income, race, education, parity, employment, and season and weighted to control for potential selection bias introduced by restricting to a sample of Live Birth. ^3^ Adjusted for all sociodemographic and lifestyle covariates which included age, smoking, season, exercise, income, race, education, alcohol, parity, aspirin, employment, vitamins, and BMI and weighted to control.

### Appendix A.3

**Table A3:** Distribution of vitamin D and Nausea/Vomiting via daily diaries for women who had a pregnancy loss versus a live birth over gestational weeks 3–4, 5–6, 7–8 weeks gestation: EAGeR Trial.

	Had a Pregnancy Loss			Live Birth		
	3–4 Weeks N = 140	5–6 Weeks N = 140	7–8 Weeks N = 140	3–4 Weeks N = 581	5–6 Weeks N = 581	7–8 Weeks N = 581
	n (%)	n (%)	n (%)	n (%)	n (%)	n (%)
**Nausea and Vomiting**						
*None*	52 (37.1)	33 (23.6)	27 (19.3)	168 (29.0)	46 (7.9)	70 (12.0)
*Nausea Only*	81 (57.9)	74 (53.0)	39.5 (27.9)	375 (64.5)	428 (73.7)	346 (59.6)
*Vomiting once per day or more than once per day*	5 (3.6)	9 (6.4)	3 (2.1)	28 (4.8)	86 (14.8)	51 (8.8)
*Missing*	2 (1.4)	24 (17.1)	71 (50.7)	10 (1.7)	21 (3.6)	114 (19.6)
